# Supplementary material for: Brain maps of general cognitive functioning: neuroimaging and neurobiological signatures
Source: Transl Psychiatry. 2025 Oct 31;15:461. doi: 10.1038/s41398-025-03617-8 (PMC12579262; doi:10.1038/s41398-025-03617-8)
Supplement: Supplementary file 1 — Supplementary Analyses [file 41398_2025_3617_MOESM1_ESM.docx]

## Supplementary analysis 1: Smoothing tolerances

The smoothing tolerance chosen for vertex-wise analyses could have an impact on the results. Noise in the data, due to registration inaccuracies, is minimized when the cortex is parcellated into larger regions (i.e., greater smoothing) but, when the cortex is parcellated into smaller regions (i.e., less smoothing), the % variance explained increases (41F^[[1]](#endnote-2)^) due to the additional information provided. Thus, at the vertex-wise level, there is a balance to be struck between the benefits of reducing noise in the data, and the problem that increasing to higher levels of smoothing will, at a point, remove fine-grained spatial information and thus reduce the spatial specificity of detected associations. Lerch and Evans (2005) analysed the effect of different smoothing tolerances on cortical thickness measurement sensitivity, and they concluded an optimal kernel size of 30 mm (42F^[[2]](#endnote-3)^, *N =* 25). Some studies use 30 mm (43F^[[3]](#endnote-4)^,44F^[[4]](#endnote-5)^), and other common choices are 5 mm (45F^[[5]](#endnote-6)^), 10 mm (46F^[[6]](#endnote-7)^,47F^[[7]](#endnote-8)^,48F^[[8]](#endnote-9)^), 15 mm (49F^[[9]](#endnote-10)^,50F^[[10]](#endnote-11)^) or 20 mm (51F^[[11]](#endnote-12)^,52F^[[12]](#endnote-13)^).

Here, we calculated the *g-*morphometry associations for each vertex-wise measure (volume, surface area, thickness, curvature and sulcal depth) for 9 smoothing kernels (0, 5, 10, 15, 20, 25, 30, 35 and 40 mm), see Supplementary Figures S4 to S6. The trade-off between noise reduction and loss of fine-grained information is clearest with standardised βs that are all in the same direction. In figure *S1_Fig1*, the trade-off can be most clearly seen in the *g-*volume estimates – the means of the standardised βs increase with increasing smoothing tolerance alongside a u-shaped trend in maximum densities of the standardised βs with increasing smoothing tolerances (as localized results are first located through noise reduction and then lost as results converge towards the total surface effect). Generally, the optimum smoothing tolerance for these types of associations across different morphometry measures appears to be between 10-20 mm (see figure *S1_Fig1B*). Therefore, the *a priori* selection of 20 fwhm for the main *g-*morphometry analyses in this paper, in line with our previous work (e.g. ^[[13]](#endnote-14)^), appears appropriate. These results may aid future choices for similar analyses.


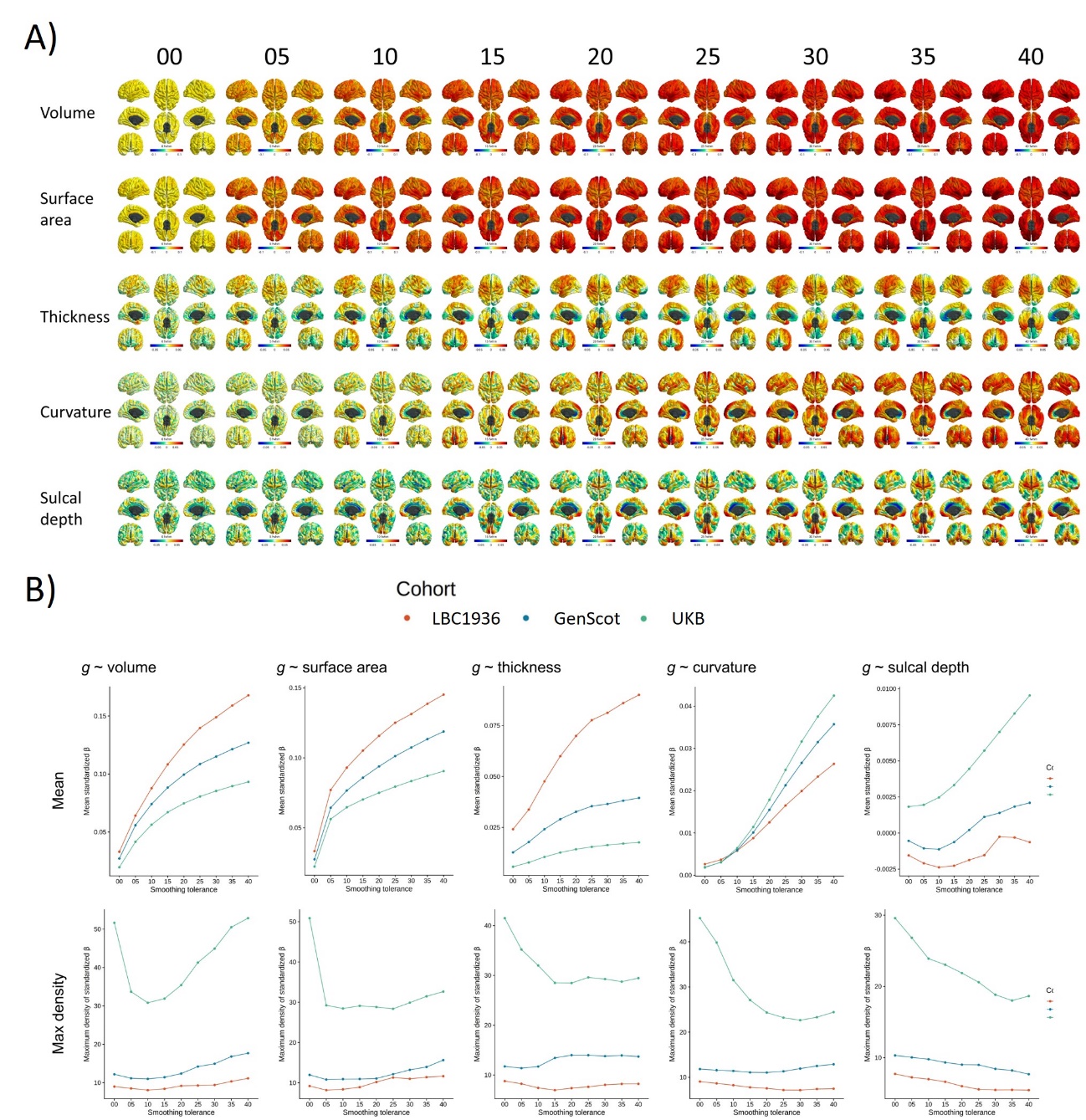


S1_Fig1 A) UKB g-morphometry associations at the 9 included smoothing tolerances. B) Summaries of the vertex-wise volume associations with g across smoothing tolerances. Top: mean standardised β at each smoothing tolerance; Bottom: maximum density of standardised β at each smoothing tolerance.

## Supplementary analysis 2: Global and subcortical volumes analyses

Although much previous work on *g-*brain associations focuses on the cortex, sub-cortical structures are becoming increasingly recognized for their associations with cognitive function. For example, reduced volumes of sub-cortical structures are reliably associated with neurodevelopmental (53F^[[14]](#endnote-15)^), psychiatric (54F^[[15]](#endnote-16)^), and neurodegenerative (55F^[[16]](#endnote-17)^) disorders. The cerebellum is also now considered to be key for several cognitive functions, although it has been historically known for its role in motor functions (56F^[[17]](#endnote-18)^). Other subcortical structures are thought to have specialised roles in cognition, such as the thalamus in memory (57F^[[18]](#endnote-19)^), and the amygdala in risk-based decision making (58F^[[19]](#endnote-20)^). Larger ventricular volumes are also indicative of poorer health, e.g., high blood pressure (59F^[[20]](#endnote-21)^) and diabetes (60F^[[21]](#endnote-22)^), and have been found to be negatively associated with performance on cognitive tests (61F^[[22]](#endnote-23)^,62F^[[23]](#endnote-24)^,63F^[[24]](#endnote-25)^).

### g ~ global and subcortical brain structures

In addition to calculating vertex-wise associations (presented in the main paper), we looked at *g-*associations for volume-based global and subcortical measures, as provided in the FreeSurfer aseg outputs. We previously provided the equivalent associations for the 68 Desikan-Killiany regions (^[[25]](#endnote-26)^). Volumes are plotted by cohort and age for global and subcortical structures in Supplementary Figure S19. First, we calculated these for each cohort, and there was strong between-cohort consistency in the relative *g-*association magnitudes across structures (all Pearson’s *r* > 0.724, all *p* < 2.2x10^16^, see Supplementary Table S14). The meta-analysed estimates (see figure *S2_Fig1* and Supplementary Table S15) show that, generally, higher *g* is associated with larger grey matter volumes, and smaller ventricular volumes. The strongest associations were for total grey matter volume and total cortex volume (β = 0.191 and β = 0.183, respectively). Other relatively large or multi-structure measures also have high positive associations: subcortical grey matter (β = 0.156), the brainstem (β = 0.148), and right and left cerebellum grey matter (β = 0.146 and β = 0.133, respectively). Smaller structures with relatively strong positive β values include the bilateral hippocampi and ventral DC (β range 0.117 to 0.148), and the anterior and posterior parts of the corpus callosum (β = 0.127 and 0.131 respectively). Negative associations are comparably weaker and show that higher *g* is associated with smaller inferior lateral ventricles (left β = -0.028 and right β = -0.057), and fewer WM hypointensities (β = -0.063). The similarity in magnitude between subcortical and cortical associations underscores the importance of subcortical structures in studies of *g.*

There was an age moderation effect for *g-*CSF associations (β = -0.011, *FDR Q* = .031), for which there were positive associations for UKB (β = 0.029, *SE =* 0.005, *p* < 2.2x10^16^) and GenScot (β = 0.072, *SE =* 0.027, *p =* .009), and a negative association in LBC1936 (β = -0.077, *SE =* 0.037, *p =* .040). This suggests that greater CSF volume in earlier life is associated with higher *g* but, in later life, increased CSF is associated with lower *g*. In younger ages, CSF volume is more strongly related to intracranial volume (ICV), but as the TBV-ICV association weakens due to atrophy, CSF becomes an important marker of atrophic differences – which can be seen in this divergence of effect sizes. There was also an age moderation effect for *g-*left pallidum associations (β = 0.011, SE = 0.003, *p* = .001); the positive association was stronger in the older cohort with a narrow age range, LBC1936 (β = 0.202, SE = 0.036, *p* = 6.^6x10-8^) than for GenScot (β = 0.057, SE = 0.028, *p* = .044) and UKB (β = 0.091, SE = 0.005, *p* < 2.^2x10-16^). However, there are noticeable differences in the magnitude of the raw volume estimations for the left pallidum between the three cohorts, which might suggest that this result is simply due to image processing differences. For all other age moderation effects for ­global and subcortical volumes with *g* associations, FDR Q > .05, see Supplementary Table S18.

### Age and sex associations with global and subcortical volumes

For age associations, only GenScot and UKB cohorts were included as the age range of LBC1936 is narrow (mean age = 72.67 years, *SD =* 0.41 years, age range = 71 to 74 years). The between-cohort correlations between the relative β magnitudes across global and subcortical volume associations with age was *r =* 0.967, *p* < 2.2x10^16^ (see figure *S2_Fig1*). Similarly, for sex associations the between-cohort correlations for sex associations with global and subcortical volumes were all high, showing good relative consistency of between-cohort effects - all r > 0.79, see Supplementary Table S14. Between-cohort age moderation effects were not calculated for these age associations, as there were only two cohorts, but there were no age moderation effects on sex-volume associations (all FDR Q > .05, see Supplementary Table S19).

With increasing age, grey matter structures decrease in volume and both ventricles and WM hypointensities increase in volume. Subcortical structures with the largest negative associations between volume and age include the accumbens areas (β = -0.431 and -0.375, left and right), hippocampi (β = -0.364 and -0.363, left and right) and thalami (β = -0.336 and -0.328, left and right), which all have larger associations than total grey matter (β = -0.319). White matter hypointensity volumes have the strongest positive associations with age of all the presently included measures (β = 0.487, *p* < .001).

All meta-analysed sex associations with global and subcortical volumes were positive – in other words, males tend to have larger volumes for all structures than females. The strongest sex-associations were with larger scale measures – e.g., TBV β = 0.466, cerebral GM β = 0.455, cerebral WM β = 0.462, subcortical GM β = 0.466. The smallest associations are for the portions of the corpus callosum (β range = 0.028 to 0.120). See Figure ii for details.

Importantly for the current main focus of this paper, the relative correlation of global and subcortical volumes by *g* and by age β estimates is r = -0.860, p = 2.86x10^13^, suggesting there is strong agreement between global and subcortical volumes that are most strongly associated with *g* and those that change the most with age. The relative correlation between global and subcortical volumes by *g* and by sex associations is r = 0.305, p = .0496, which does not reach the significance threshold.





S2_fig1 Associations between g, age and sex and global and subcortical structures. A) The meta-analysed subcortical estimates mapped to the brain (top A: β estimates, middle A: log FDR Q values, bottom A: FDR Q values). For β estimates and log Q values, the colour scale limits are set to the maximum of the vertex-wise or present results for each independent variable. For age and sex, some p values were estimated at 0, with an R print limit of 1000. For these values, for log Q values, they are set at the limit of the scale which is -704.3. These associations are shown on the ICBM 2009b non-linear asymmetric mni template brain. B) Forest plot showing standardised β estimates for each cohort and meta-analysed estimates.

## Supplementary Analysis 3: Do regional *g-*morphometry associations differ by sex?

Although we controlled for sex in the main analyses, we decided to additionally conduct a supplementary analysis to test whether regional *g-*morphometry associations differ by sex. Across global and subcortical analyses, there were high correlations between the β values derived from males and those from females (all *r* > 0.80, all *p* < .0001, see Figure *S3_Fig1*). However, for vertex-wise analyses, whilst there were similarly high correlations for male and female groups for UKB (all *r* > 0.753), correlations tended to be smaller for both LBC1936 and GenScot (see Table i). There are several possible explanations for these findings. It may be that smaller sample sizes lead to less stable association patterns (N_maleUKB_ = 17393, N_femaleUKB_ = 19358, N_maleGenScot_ = 408, N_femaleGenScot_ = 606, N_maleLBC1936_ = 294, N_femaleLBC1936_ = 328). Additionally, the LBC1936 is a narrow-age cohort (mean age = 72.67 years, *SD =* 0.41) and the GenScot imaging sample was selected for depression – sex differences in the way that brain morphometry relates to *g* might be more pronounced in these samples than in the more generally sampled UKB. If meaningful sex differences exist in the general population, we would have expected to see them in the UKB sample.


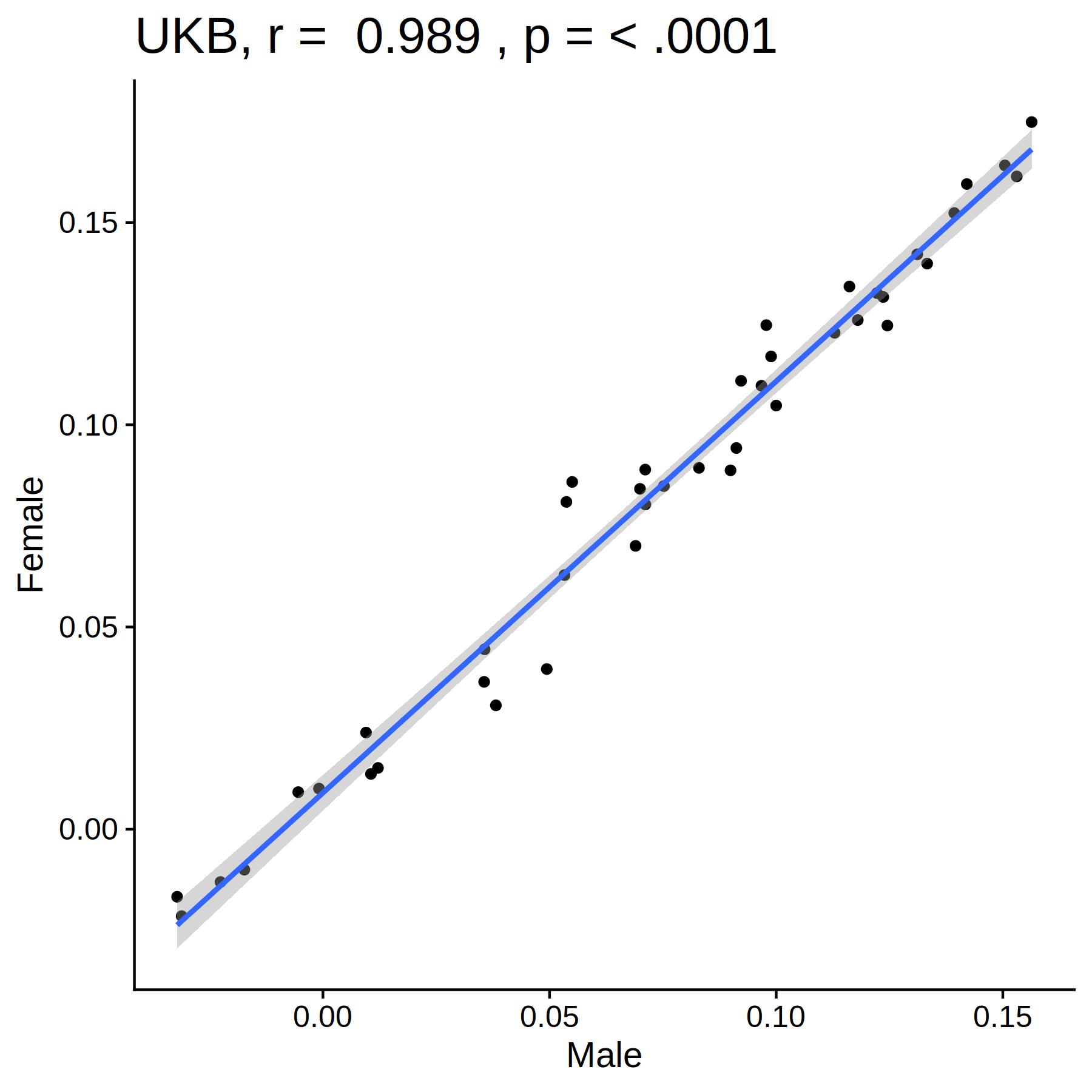

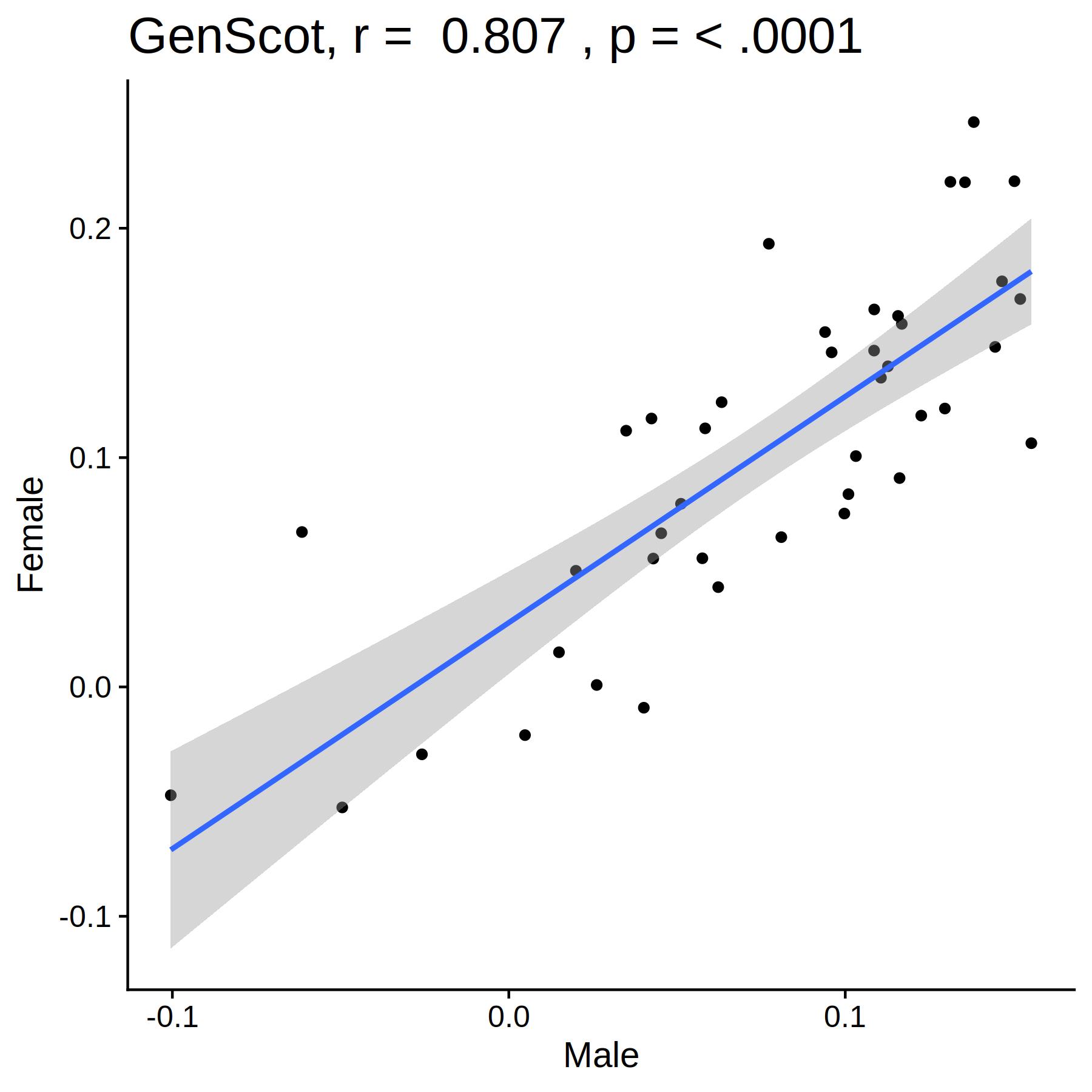

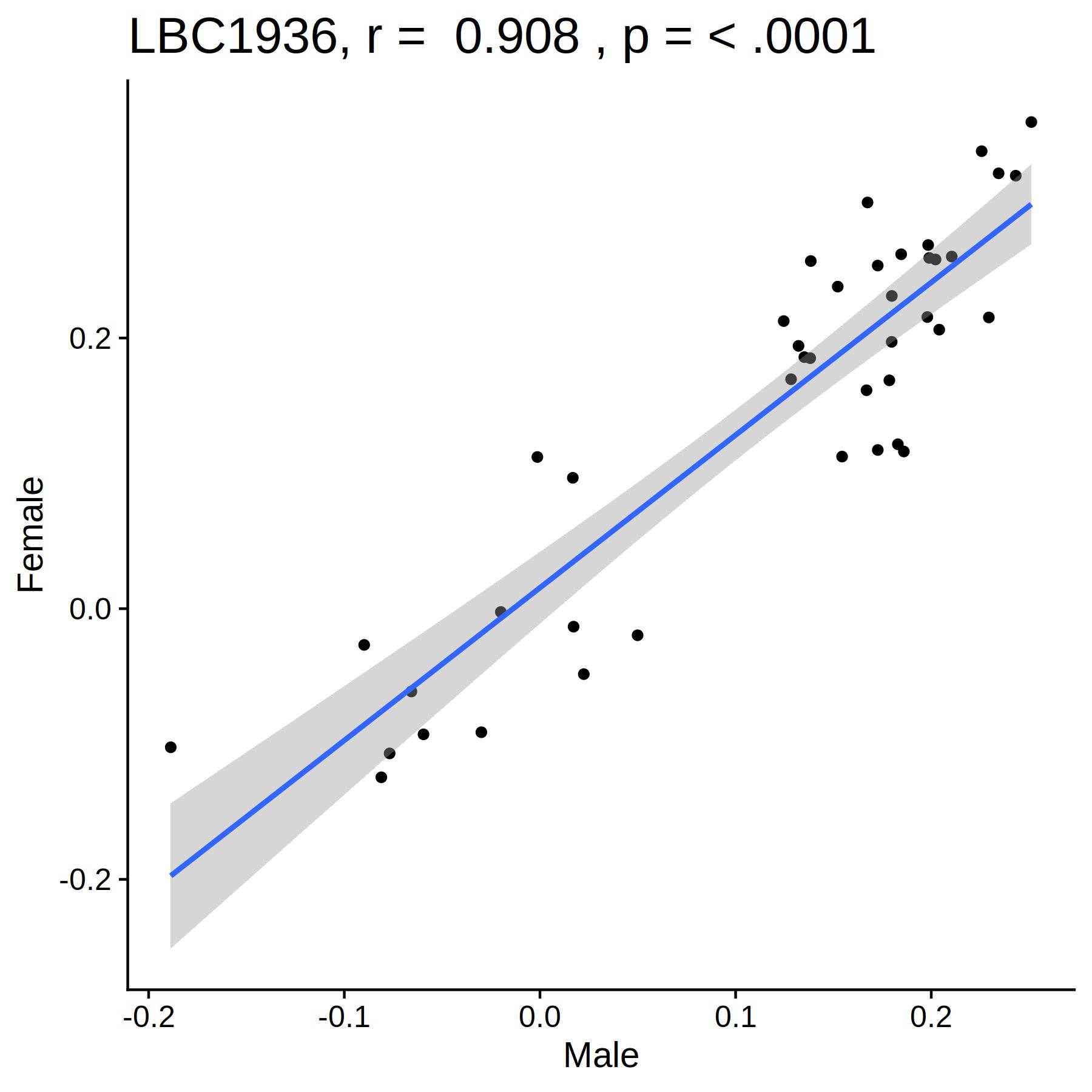


*S3_Fig1 Scatter plots showing correlations between g-volume estimates for global and subcortical structures for males against females.*

*Table i* Correlations between male and female *g-*morphometry profiles in each cohort (Pearson’s *r*).

|  | LBC1936 | GenScot | UKB |
| --- | --- | --- | --- |
| Volume | 0.281 | 0.152 | 0.808 |
| Surface area | 0.368 | 0.247 | 0.847 |
| Thickness | 0.307 | 0.349 | 0.753 |
| Curvature | 0.374 | 0.172 | 0.831 |
| Sulcal depth | 0.536 | 0.426 | 0.844 |

## Supplementary Analysis 4: Do *g-*correlations with neurobiological maps differ between the fsaverage164k/fsaverage 10k/Schaefer 1000 atlases?


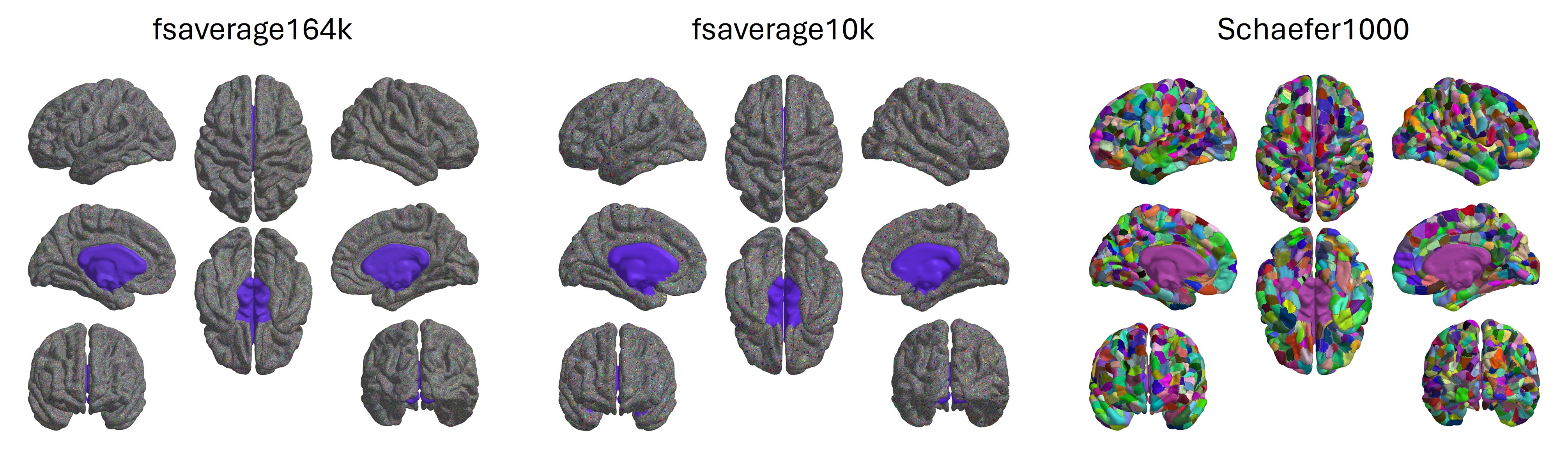


*S4_Fig1 The three atlases tested here: fsaverage164k (as in the main analysis), total vertices = 327,684; fsaverage10k, total vertices = 20,484; and Schaefer 1000, total regions = 1000.*

With thanks to comment from an anonymous reviewer, we ran the *g-*correlations with neurobiological maps with the maps registered to 1) the fsaverage 10k surface, and 2) the Schaefer1000 atlas (see figure *S4_Fig1*). This analysis shows that the correlations we obtain were not strongly affected by upsampling the data to the fsaverage 164k surface, and they were not strongly affected by the atlas.

To get all the maps in the fsaverage 10k space, we downsampled the maps that were originally created/sourced in fsaverage164k space -- this included the *g-*morphometry maps, allometric scaling map, mean surface area and thickness, the principal component of metabolism and the similarity eigenvectors from BigBrainWarp (microstructural gradients 1 and 2, cytoarchitectural gradients 1 and 2, and functional gradients 1 and 2). For maps that did not originate in the fsaverage164k space, we transformed them from their original space to the fsaverage 10k space, using the transforms function in neuromaps. We applied 5 mm FWHM smoothing to the neurotransmitter receptor maps, compared to 20 mm FWHM for the 164k space, reflecting the approximately 15-fold difference in spatial resolution between the two maps.

To get the maps in the Schaefer atlas space, we used the fsaverage 164k maps, which we report in the main analyses, and parcellated them to the Schaefer 1000 atlas using the .annot files (the 7-Network version), provided in Thomas Yeo’s Lab’s Computation Brain Imaging Group github repository <https://github.com/ThomasYeoLab/CBIG>. We took the mean values of all of the vertices included in each of the 1000 parcels, resulting in 1000 values for each cortical map.

The correlations between *g-*morphometry profiles and neurobiological profiles were very similar for each of the three atlases (r range = 0.991, between fsaverage164k and Schaefer1000 for *g-*thickness, sulcal depth, to 0.999, between fsaverage164k and fsaverage10k for *g-*volume), see Figure S4_Fig2 and S4_Fig3). Therefore, the spatial correlations between different cortical profiles appear to be robust across different atlases.


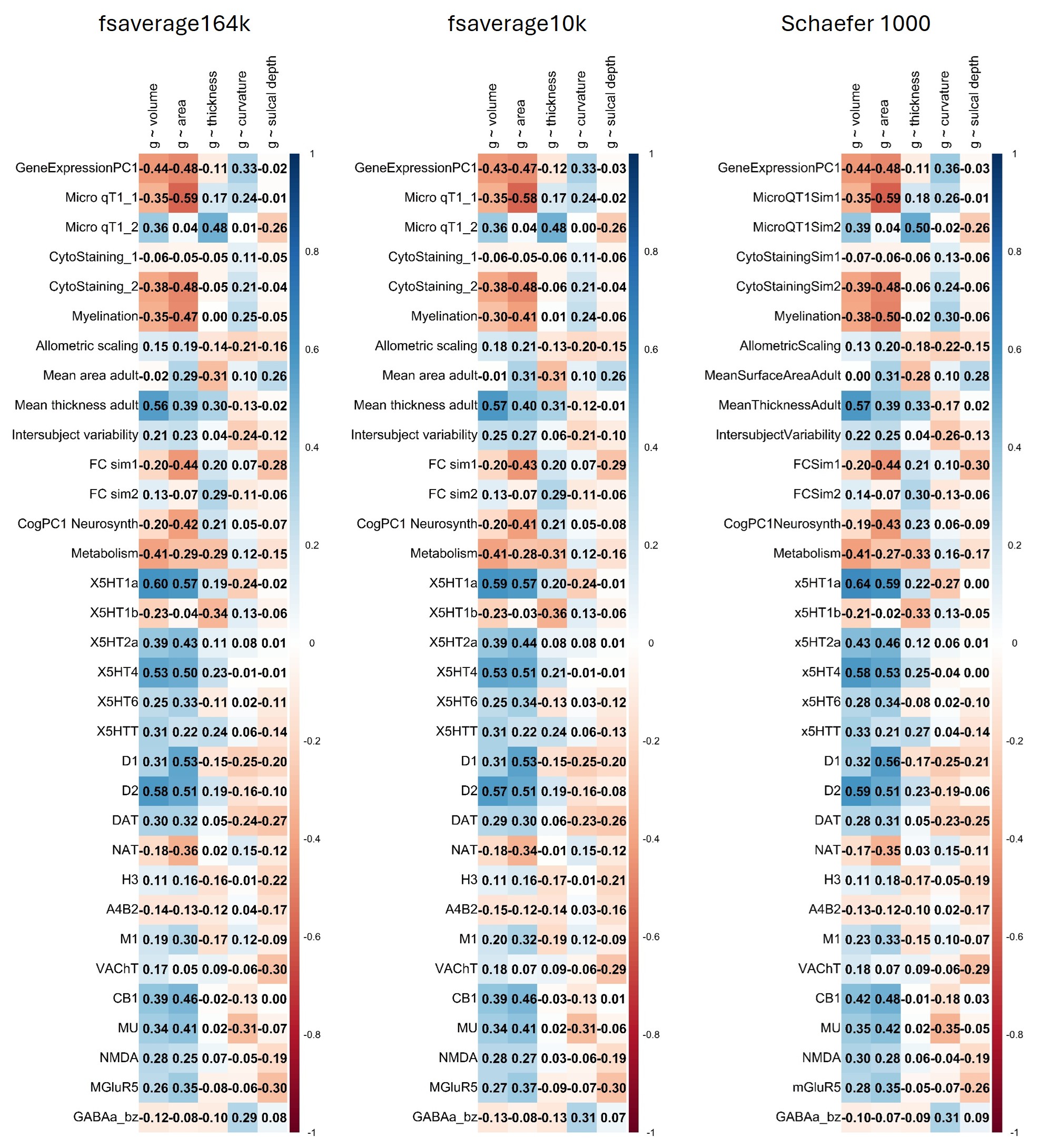


*S4_Fig2 The correlations between g and neurobiological profiles for the fsaverage 164k, fsaverage 10k and Schaefer 1000 cortical spaces.*


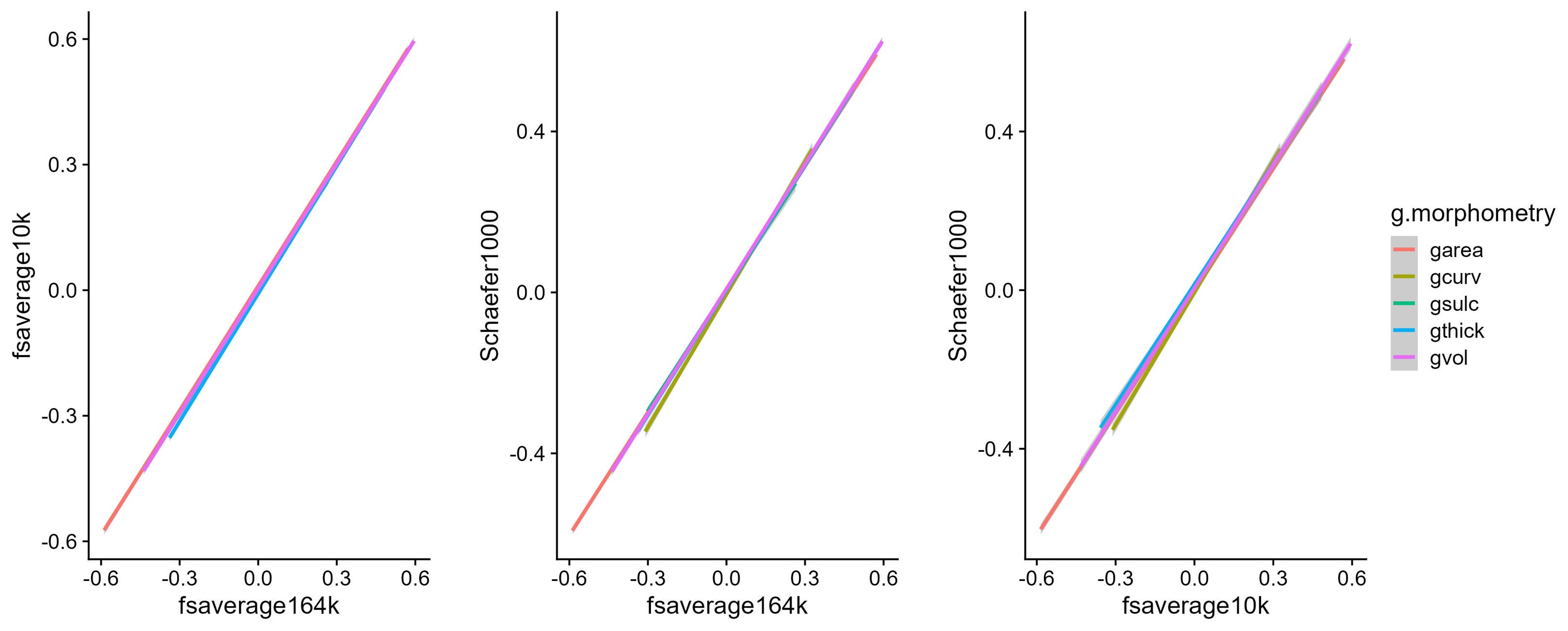


*S4_Fig3 Correlations between different atlas types.*

Given that we then had our brain profiles in three different atlas spaces, we took the opportunity to determine how spin test results differ for these different atlases of varying parcellation size. The results of the spin tests for each of the three atlases for 48x48 maps (g-morphometry x5, age-morphometry x5, sex-morphometry x5, 33 neurobiological maps) are available in the Supplementary Tabular Data File. The results shown in Figure *S4_Fig4* show that the spin test is less conservative with larger parcellations (the Schaefer1000 equal size parcellation atlas, compared to the fsaverage164k and fsaverage10k atlases). There were N = 368 correlations with *p* < .05 for fsaverage164k, N = 378 for fsaverage10k, and N = 513 for the Schaefer1000 atlas. There was also an increased number of significant values in the Benjamini-Hochberg p_spin correction with increasing coarseness of parcellation: there were N = 246 correlations with BH p_spin < .05 for fsaverage 164k, N = 255 for fsaverage10k, and N = 384 for the Schaefer1000 atlas. Note that the fsaverage164k and fsaverage10k surfaces were smoothed according to their relative sizes (20 fwhm and 5 fwhm, respectively, given the 15-fold difference between them). Therefore, the spatial fidelity is comparable between the two map types, which likely accounts for their similar spin test results. These results might be partially explained by a number of things including a higher signal-to-noise ratio with larger parcels (when the maps are spun).


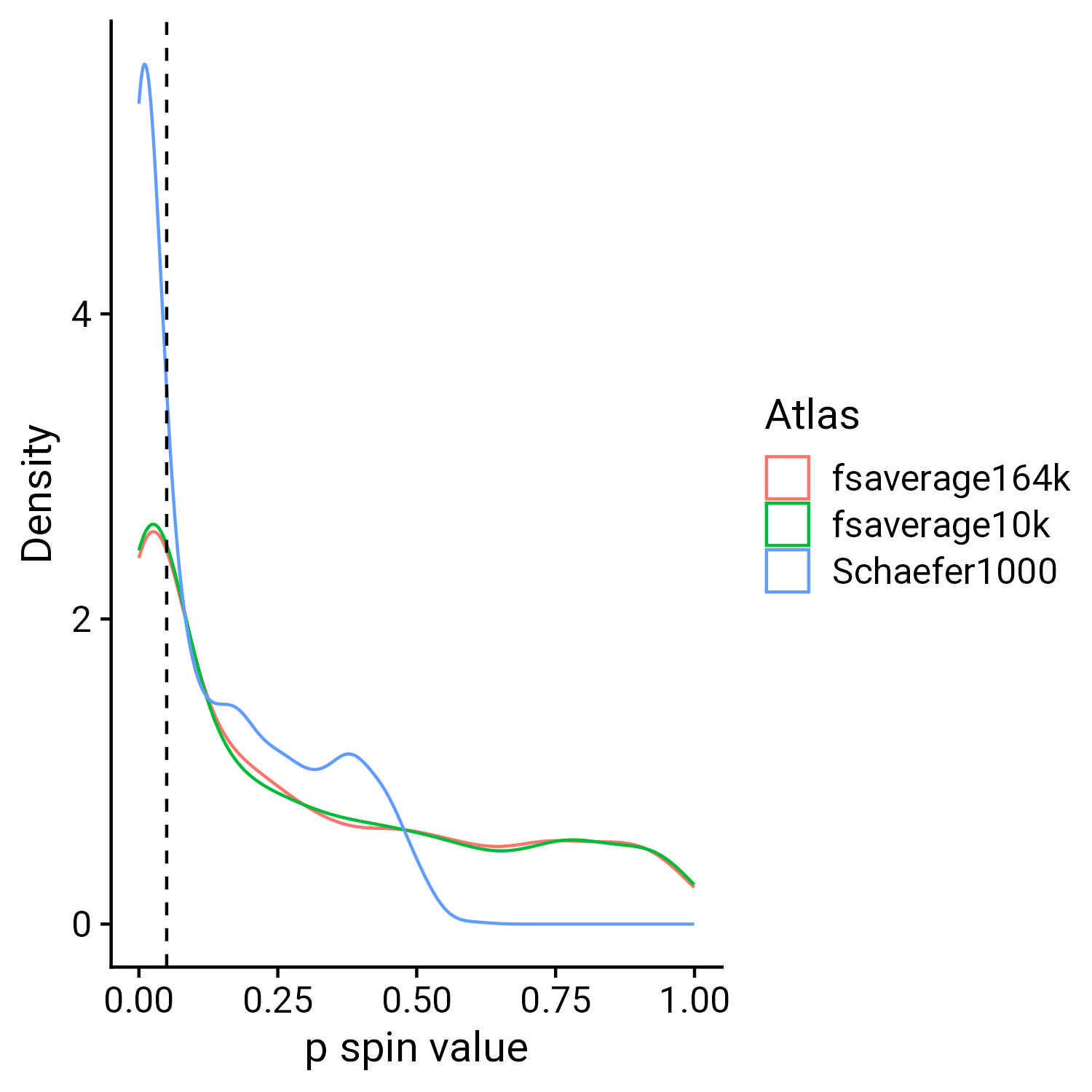

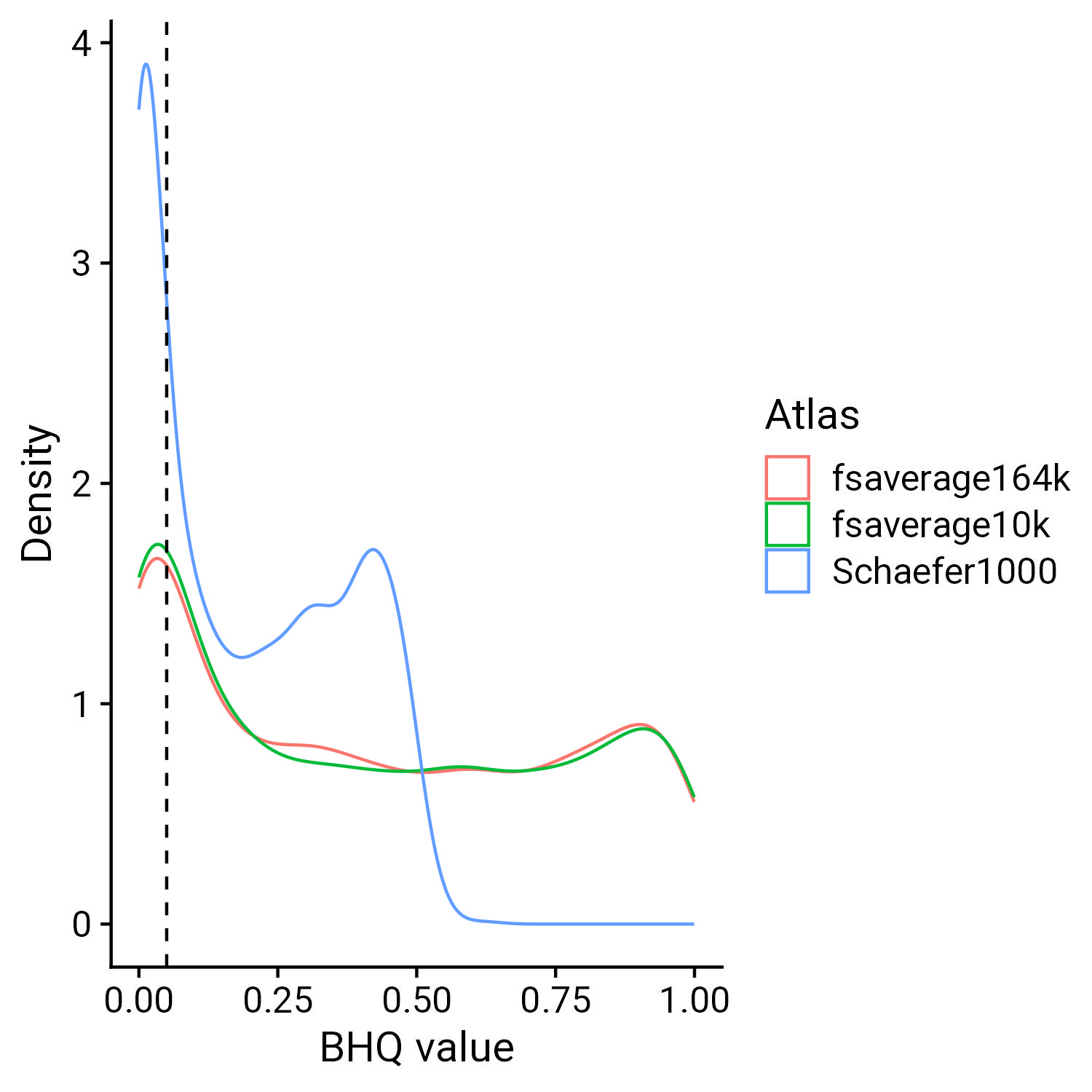


*S4_Fig4 Density plots of Left: p spin values, and Right Benjamini-Hochberg corrected BH_pp_spin values for the three atlases (fsaverage164k, fsaverage10k, and Schaefer1000), across 48x48 spatial correlations (g-morphometry maps x5, age-morphometry maps x5, sex-morphometry maps x5, and the 33 neurobiological and neurostructural maps).*

## Supplementary Analysis 5: Within-region correlations are not led by region size

An anonymous reviewer was concerned that the differences in within-region correlations that we found might be explainable by parcel size, as the regions within the Desikan-Killiany atlas have variable volumes. We analysed the correlations between our within-region correlations of *g-*morphometry associations and mean volume of the 68 Desikan Killiany regions (previously meta-analysed using the same three cohorts as the current paper (LBC1936, GenScot and UKB) and provided in the Supplementary Information in Moodie et al. (2024)25). The within-region correlations included those between *g-*morphometry and age-morphometry, sex-morphometry and the 33 neurobiological and neurostructural profiles. The results (shown in Figure *S5_Fig1*) show that there were no FDR significant (α < .05) correlations (the FDR method was applied per *g-*morphometry measure). Therefore, the within-region correlations do not seem to be led by parcel size. These findings are consistent with the hypothesis that there are meaningful within-region correlations differences, which could be masked by cortex-wide correlations. We would suggest that it is important to bear this in mind when interpreting cortex-wide correlations, and to perform within-region correlations when it fits with hypotheses.


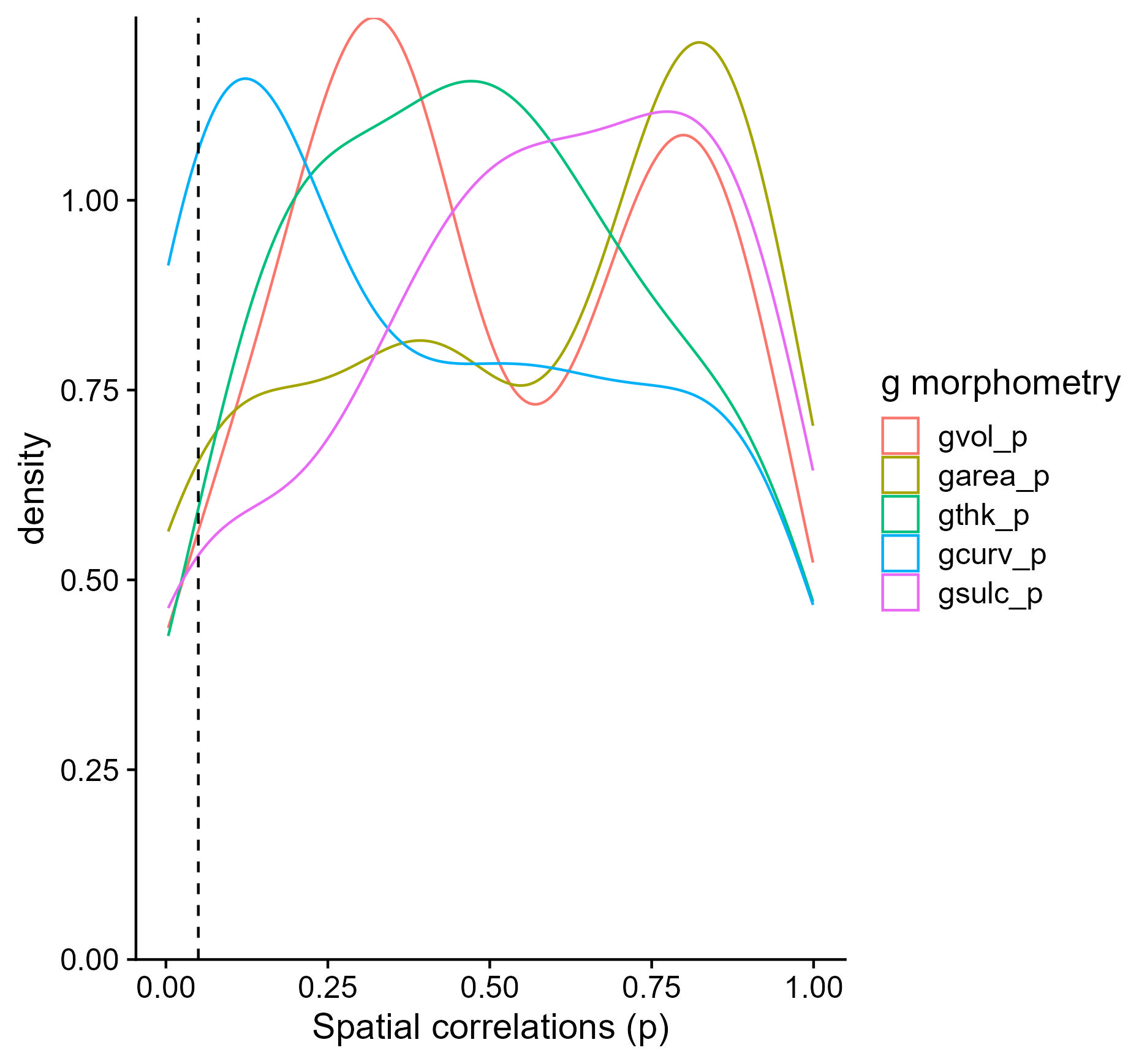

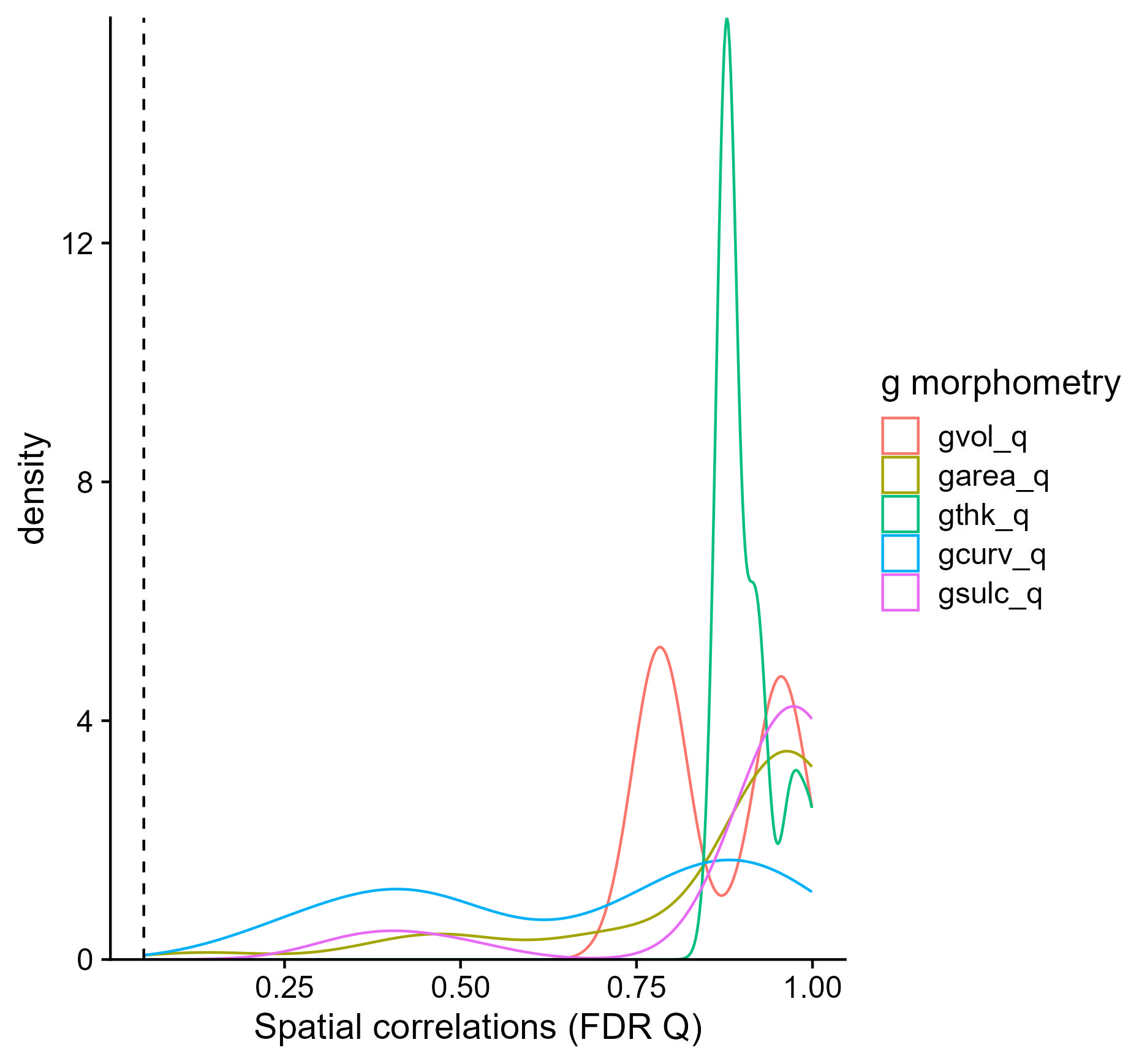


*S5_Fig1 Density graphs showing (Left) p values and (Right) FDR Q values for correlations between within-region correlations and mean volume size of the 68 Desikan Killiany regions (previously calculated and provided in the Supplementary Tabular Data File in Moodie et al. (2024).*

## Supplementary Analysis 6: Spatial correlations between morphometry measures

Whilst there is shared regional patterning across mean morphometry measures (see Figure S6_Fig1), we chose to calculate *g-*morphometry associations on each morphometry measure separately (rather than perform dimension reduction) because the main aim of our study was to look at spatial correlations between *g-*morphometry patterns and neurobiological/neurostructural patterns. Different morphometric measures are likely to reflect distinct underlying biological mechanisms, which may arise from different neurodevelopmental, genetic and microstructural processes ^[[26]](#endnote-27)^. We therefore deemed it important to test the neurobiological correlations for each *g-*morphometry map separately. Figure S6_Fig1 shows that the correlations between the morphometry means differ from the correlations of the correlations between *g-*morphometry and neurobiological/neurostructural profiles. For example, correlations of *g-*morphometry and neurobiological profiles, *g-*curvature is much more strongly related to *g-*volume and particularly *g-*surface area (right side panel of S6_Fig1) than mean curvature is to mean volume and mean area (left side panel of S6_Fig1).


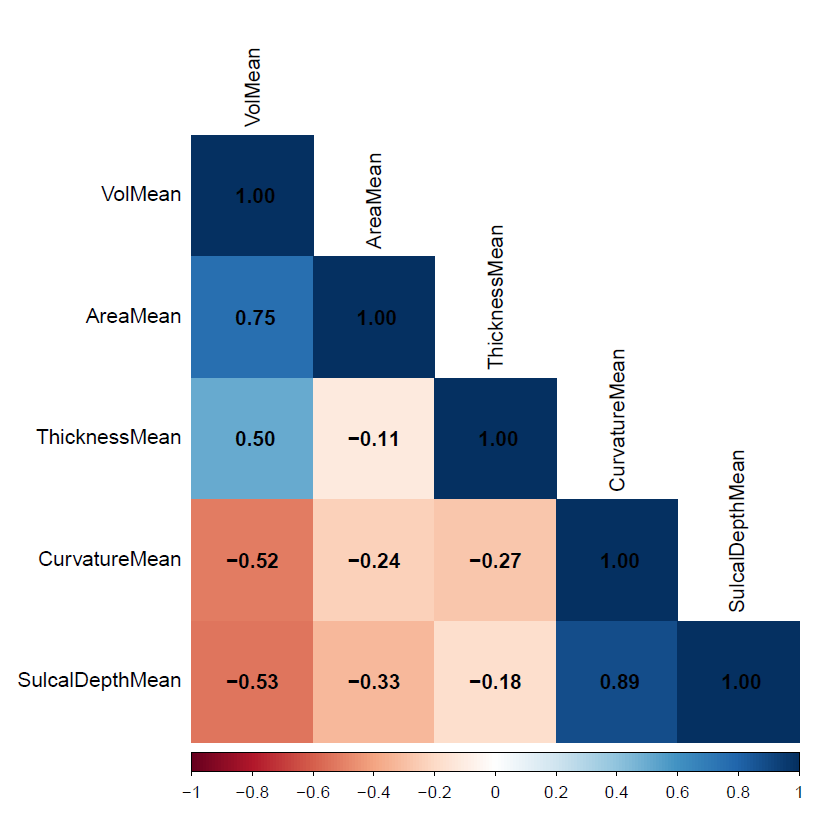




*S6_Fig1 Left: correlations between vertex-wise mean morphometry measures; Right: correlations of the correlations between g-morphometry with neurobiological/neurostructural profiles for each of the 5 morphometry measures.*

## Supplementary Analysis 7: Correlations between neurotransmitter density maps and mean morphometry measures

An anonymous reviewer brought our attention to the issue of partial volume effects being potentially exacerbated by transforming PET images to the fsaverage surface, resulting in an image that is heavily biased by brain curvature. To check whether this was the case in the neurotransmitter maps we used here, we correlated the maps with the mean sulcal depth. We also correlated the maps with the means of the other morphometry measures (volume, surface area, thickness and curvature) to determine whether there was a special relationship between these maps and mean sulcal depth.

The results (see Figure S7_Fig1) show that the spatial correlations between neurotransmitter maps and mean sulcal depth (absolute mean r = |0.188|) were no larger than their spatial correlations with other mean morphometry measures (respectively, for volume, surface area, thickness and curvature, mean r = |0.236|, mean r = |0.186|, mean r = |0.367|, and mean r = |0.19|). Furthermore, the patterning of correlations with the 5 mean morphometry measures were not consistent across neurotransmitters (see Figure S7_Fig2). This suggests that the morphometry means do not systematically impact the neurotransmitter cortical surface maps, due to an issue with transformations between volumetric and surface space.

Note we smoothed the PET volumetric images by 20 mm FWHM after they were transformed to the surface, which could explain why we do not see substantial partial volume effects in our data. We did not test unsmoothed data or data different smoothing levels, which might show partial volume effects in these neurotransmitter data.


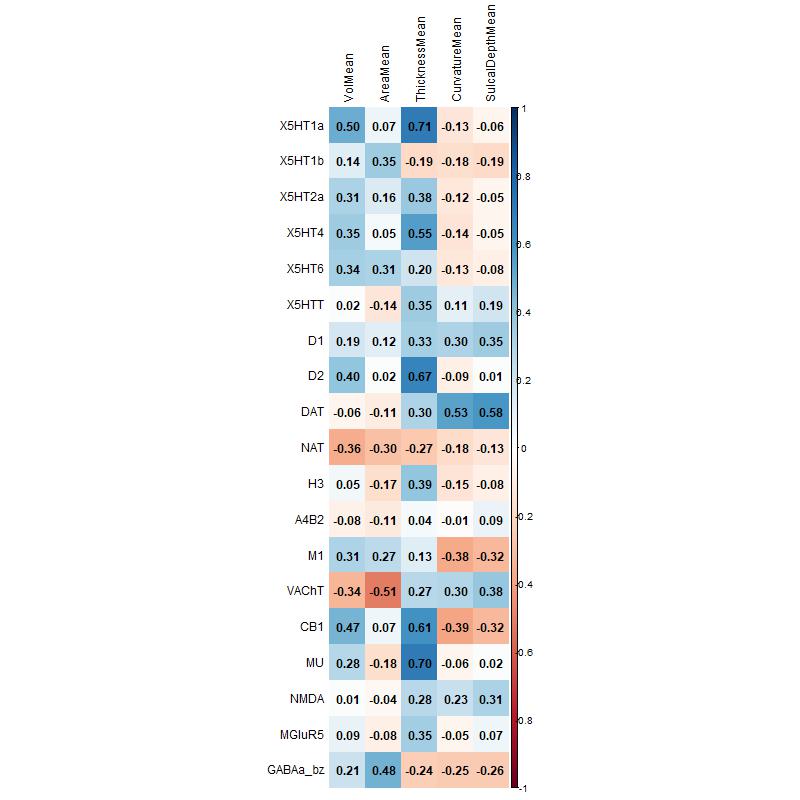

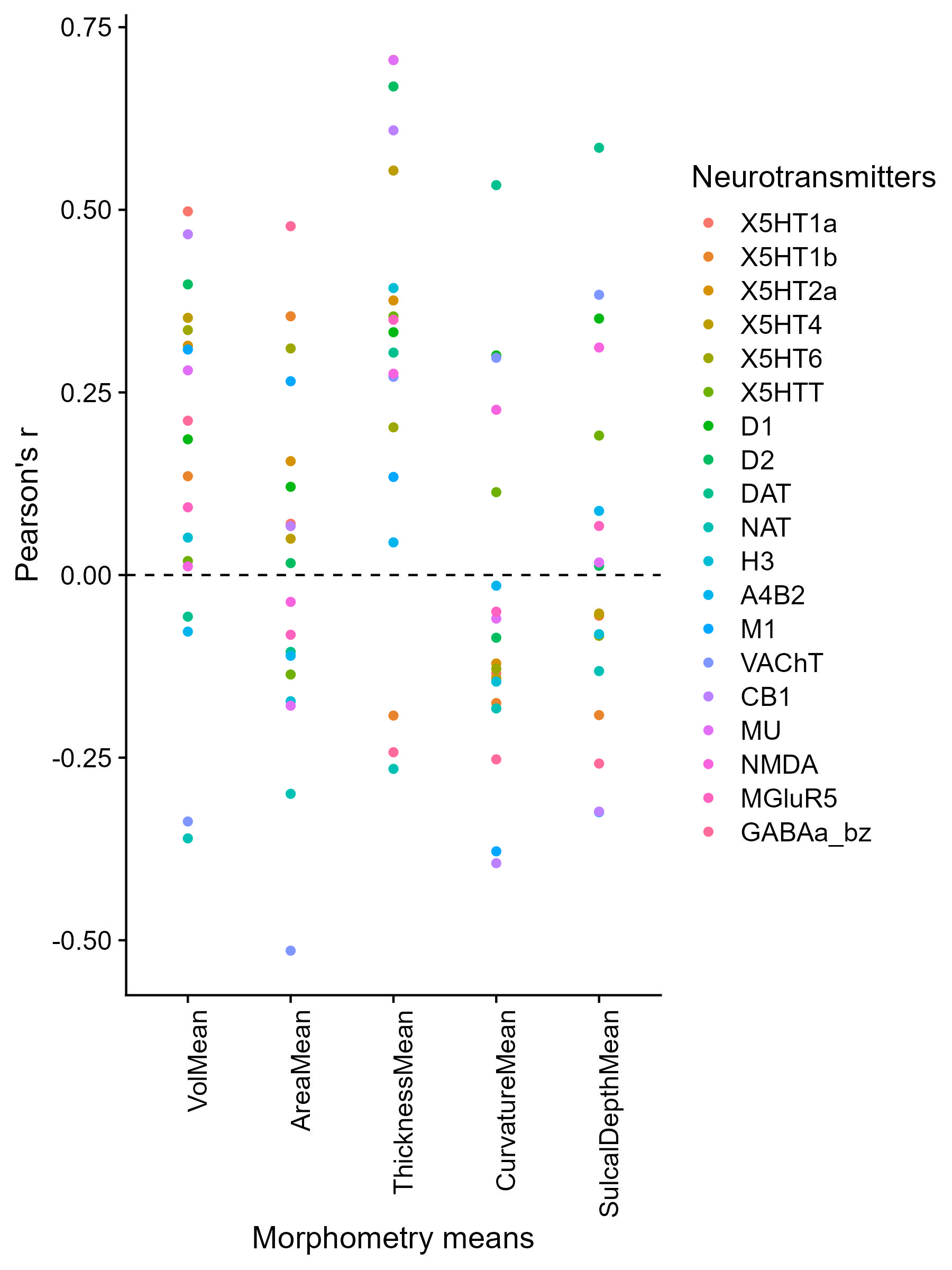


*S7_Fig1 Spatial correlations between neurotransmitter maps and mean morphometry maps. Left: Correlation table; Right: plot showing the correlation magnitudes for each morphometry mean across neurotransmitter receptor maps.*


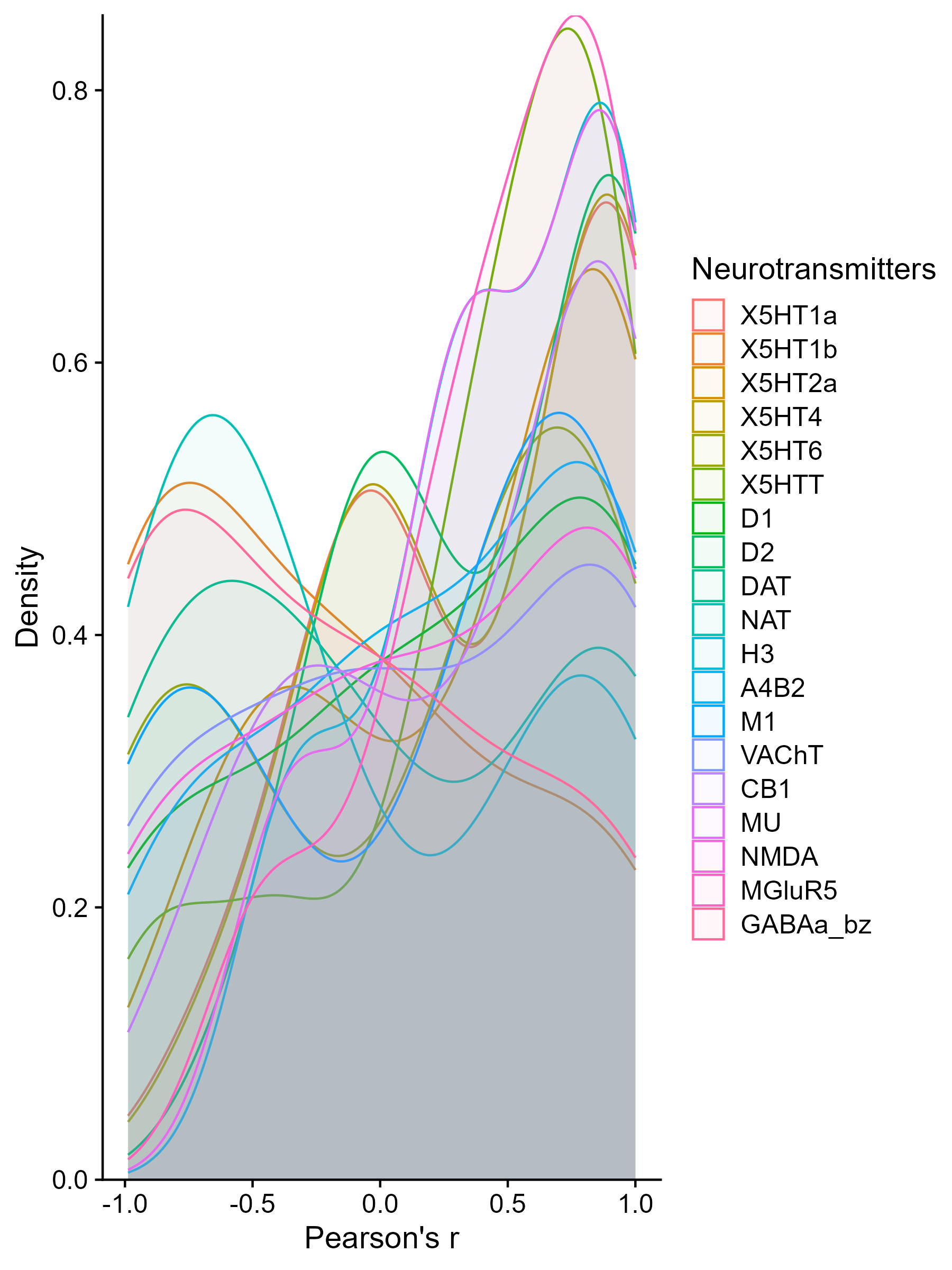


*S7_Fig2 Density map of correlations between neurotransmitters of their correlations with the 5 mean morphometry measures (mean volume, surface area, thickness, curvature, and sulcal depth) (correlation of correlations). This figure suggests that the morphometry means do not systematically impact the neurotransmitter cortical surface maps, due to an issue with transformations between volumetric and surface space.*

## References

1. Fürtjes, A. E., Cole, J. H., Couvy-Duchesne, B., & Ritchie, S. J. (2023). A quantified comparison of cortical atlases on the basis of trait morphometricity. Cortex; a journal devoted to the study of the nervous system and behavior, 158, 110–126. https://doi.org/10.1016/j.cortex.2022.11.001 [↑](#endnote-ref-2)
2. Lerch, J. P., & Evans, A. C. (2005). Cortical thickness analysis examined through power analysis and a population simulation. *NeuroImage*, *24*(1), 163–173. https://doi.org/10.1016/j.neuroimage.2004.07.045 [↑](#endnote-ref-3)
3. Buchy, L., Barbato, M., Makowski, C., Bray, S., MacMaster, F. P., Deighton, S., & Addington, J. (2017). Mapping structural covariance networks of facial emotion recognition in early psychosis: A pilot study. *Schizophrenia research*, *189*, 146–152. https://doi.org/10.1016/j.schres.2017.01.054 [↑](#endnote-ref-4)
4. Chung, M. K., Worsley, K. J., Robbins, S., Paus, T., Taylor, J., Giedd, J. N., Rapoport, J. L., & Evans, A. C. (2003). Deformation-based surface morphometry applied to gray matter deformation. *NeuroImage*, *18*(2), 198–213. https://doi.org/10.1016/s1053-8119(02)00017-4 [↑](#endnote-ref-5)
5. Tadayon, E., Pascual-Leone, A., & Santarnecchi, E. (2020). Differential Contribution of Cortical Thickness, Surface Area, and Gyrification to Fluid and Crystallized Intelligence. *Cerebral cortex (New York, N.Y. : 1991)*, *30*(1), 215–225. https://doi.org/10.1093/cercor/bhz082 [↑](#endnote-ref-6)
6. Nho, K., Risacher, S. L., Crane, P. K., DeCarli, C., Glymour, M. M., Habeck, C., Kim, S., Lee, G. J., Mormino, E., Mukherjee, S., Shen, L., West, J. D., Saykin, A. J., & Alzheimer’s Disease Neuroimaging Initiative--ADNI (2012). Voxel and surface-based topography of memory and executive deficits in mild cognitive impairment and Alzheimer's disease. *Brain imaging and behavior*, *6*(4), 551–567. https://doi.org/10.1007/s11682-012-9203-2 [↑](#endnote-ref-7)
7. Lamballais, S., & Muetzel, R. L. (2021). QDECR: A Flexible, Extensible Vertex-Wise Analysis Framework in R. *Frontiers in neuroinformatics*, *15*, 561689. https://doi.org/10.3389/fninf.2021.561689 [↑](#endnote-ref-8)
8. Tadayon, E., Pascual-Leone, A., & Santarnecchi, E. (2020). Differential Contribution of Cortical Thickness, Surface Area, and Gyrification to Fluid and Crystallized Intelligence. *Cerebral cortex (New York, N.Y. : 1991)*, *30*(1), 215–225. https://doi.org/10.1093/cercor/bhz082 [↑](#endnote-ref-9)
9. Holla, B., Bharath, R. D., Venkatasubramanian, G., and Benegal, V. (2019) Altered brain cortical maturation is found in adolescents with a family history of alcoholism, *Addiction Biology*, 24, 835– 845. doi: <https://doi.org/10.1111/adb.12662>. [↑](#endnote-ref-10)
10. Wang, Y., Jiang, Y., Lu, H., Tian, W., Li, P., Xu, K., Fan, M., Zhao, X., Dong, Q., Jin, L., Chen, J., Cui, M., & Chen, X. (2022). Cross-sectional associations between cortical thickness and independent gait domains in older adults. *Journal of the American Geriatrics Society*, *70*(9), 2610–2620. <https://doi.org/10.1111/jgs.17840> [↑](#endnote-ref-11)
11. Cox, S. R., Lyall, D. M., Ritchie, S. J., Bastin, M. E., Harris, M. A., Buchanan, C. R., Fawns-Ritchie, C., Barbu, M. C., de Nooij, L., Reus, L. M., Alloza, C., Shen, X., Neilson, E., Alderson, H. L., Hunter, S., Liewald, D. C., Whalley, H. C., McIntosh, A. M., Lawrie, S. M., Pell, J. P., … Deary, I. J. (2019). Associations between vascular risk factors and brain MRI indices in UK Biobank. European heart journal, 40(28), 2290–2300. https://doi.org/10.1093/eurheartj/ehz100 [↑](#endnote-ref-12)
12. Cox, S. R., Dickie, D. A., Ritchie, S. J., Karama, S., Pattie, A., Royle, N. A., Corley, J., Aribisala, B. S., Valdés Hernández, M., Muñoz Maniega, S., Starr, J. M., Bastin, M. E., Evans, A. C., Wardlaw, J. M., & Deary, I. J. (2016). Associations between education and brain structure at age 73 years, adjusted for age 11 IQ. Neurology, 87(17), 1820–1826. https://doi.org/10.1212/WNL.0000000000003247 [↑](#endnote-ref-13)
13. Cox, S. R., Dickie, D. A., Ritchie, S. J., Karama, S., Pattie, A., Royle, N. A., Corley, J., Aribisala, B. S., Valdés Hernández, M., Muñoz Maniega, S., Starr, J. M., Bastin, M. E., Evans, A. C., Wardlaw, J. M., & Deary, I. J. (2016). Associations between education and brain structure at age 73 years, adjusted for age 11 IQ. *Neurology*, *87*(17), 1820–1826. https://doi.org/10.1212/WNL.0000000000003247 [↑](#endnote-ref-14)
14. Ecker, C., Bookheimer, S. Y., & Murphy, D. G. (2015). Neuroimaging in autism spectrum disorder: Brain structure and function across the lifespan. Lancet Neurology, 14, 1121–1134. https://doi.org/10.1016/S1474-4422(15)00050-2. [↑](#endnote-ref-15)
15. Hibar, D. P., Westlye, L. T., van Erp, T. G., Rasmussen, J., Leonardo, C. D., Faskowitz, J., … Andreassen, O. A. (2016). Subcortical volumetric abnormalities in bipolar disorder. Molecular Psychiatry, 21, 1710–1716. https://doi.org/10.1038/mp.2015.227 [↑](#endnote-ref-16)
16. Watson, R., Colloby, S. J., Blamire, A. M., & O'Brien, J. T. (2016). Subcortical volume changes in dementia with Lewy bodies and Alzheimer's disease. A comparison with healthy aging. *International psychogeriatrics*, *28*(4), 529–536. https://doi.org/10.1017/S1041610215001805 [↑](#endnote-ref-17)
17. Buckner R. L. (2013). The cerebellum and cognitive function: 25 years of insight from anatomy and neuroimaging. *Neuron*, *80*(3), 807–815. https://doi.org/10.1016/j.neuron.2013.10.044 [↑](#endnote-ref-18)
18. Vetere, G., Xia, F., Ramsaran, A. I., Tran, L. M., Josselyn, S. A., & Frankland, P. W. (2021). An inhibitory hippocampal-thalamic pathway modulates remote memory retrieval. *Nature neuroscience*, *24*(5), 685–693. https://doi.org/10.1038/s41593-021-00819-3 [↑](#endnote-ref-19)
19. Takeuchi, H., Tsurumi, K., Murao, T., Mizuta, H., Murai, T., & Takahashi, H. (2019). Amygdala volume is associated with risky probability cognition in gambling disorder. *Addiction biology*, *24*(4), 802–810. https://doi.org/10.1111/adb.12640 [↑](#endnote-ref-20)
20. Goldstein, I. B., Bartzokis, G., Guthrie, D., & Shapiro, D. (2002). Ambulatory blood pressure and brain atrophy in the healthy elderly. *Neurology*, *59*(5), 713–719. https://doi.org/10.1212/wnl.59.5.713 [↑](#endnote-ref-21)
21. Ferguson, S. C., Blane, A., Wardlaw, J., Frier, B. M., Perros, P., McCrimmon, R. J., & Deary, I. J. (2005). Influence of an early-onset age of type 1 diabetes on cerebral structure and cognitive function. *Diabetes care*, *28*(6), 1431–1437. https://doi.org/10.2337/diacare.28.6.1431 [↑](#endnote-ref-22)
22. Evans, M. C., Barnes, J., Nielsen, C., Kim, L. G., Clegg, S. L., Blair, M., Leung, K. K., Douiri, A., Boyes, R. G., Ourselin, S., Fox, N. C., & Alzheimer's Disease Neuroimaging Initiative (2010). Volume changes in Alzheimer's disease and mild cognitive impairment: cognitive associations. *European radiology*, *20*(3), 674–682. https://doi.org/10.1007/s00330-009-1581-5 [↑](#endnote-ref-23)
23. Apostolova, L. G., Babakchanian, S., Hwang, K. S., Green, A. E., Zlatev, D., Chou, Y. Y., DeCarli, C., Jack, C. R., Jr, Petersen, R. C., Aisen, P. S., Cummings, J. L., Toga, A. W., & Thompson, P. M. (2013). Ventricular enlargement and its clinical correlates in the imaging cohort from the ADCS MCI donepezil/vitamin E study. *Alzheimer disease and associated disorders*, *27*(2), 174–181. https://doi.org/10.1097/WAD.0b013e3182677b3d [↑](#endnote-ref-24)
24. Ferrarini, L., Palm, W. M., Olofsen, H., van der Landen, R., Jan Blauw, G., Westendorp, R. G., Bollen, E. L., Middelkoop, H. A., Reiber, J. H., van Buchem, M. A., & Admiraal-Behloul, F. (2008). MMSE scores correlate with local ventricular enlargement in the spectrum from cognitively normal to Alzheimer disease. *NeuroImage*, *39*(4), 1832–1838. https://doi.org/10.1016/j.neuroimage.2007.11.003 [↑](#endnote-ref-25)
25. Moodie, J. E., Harris, S. E., Harris, M. A., Buchanan, C. R., Davies, G., Taylor, A., Redmond, P., Liewald, D. C. M., Valdés Hernández, M. C., Shenkin, S., Russ, T. C., Muñoz Maniega, S., Luciano, M., Corley, J., Stolicyn, A., Shen, X., Steele, D., Waiter, G., Sandu, A.-L., … Cox, S. R. (2024). General and specific patterns of cortical gene expression as spatial correlates of complex cognitive functioning. *Human Brain Mapping*, 45(4), e26641. [**https://doi.org/10.1002/hbm.26641**](https://doi.org/10.1002/hbm.26641) [↑](#endnote-ref-26)
26. Bethlehem, R.A.I., Seidlitz, J., White, S.R. et al. Brain charts for the human lifespan. Nature **604**, 525–533 (2022). https://doi.org/10.1038/s41586-022-04554-y [↑](#endnote-ref-27)
